# Knockdown of Ephrin-A5 Expression by 40% Does not Affect Motor Axon Growth or Migration into the Chick Hindlimb

**DOI:** 10.3390/ijms12128362

**Published:** 2011-11-29

**Authors:** Robert S. Winning, Catherine E. Krull

**Affiliations:** 1 Biology Department, Eastern Michigan University, Ypsilanti, MI 48197, USA; 2 The Department of Biologic and Materials Sciences, School of Dentistry, University of Michigan, 5211 Dental, 1011 N. University Avenue, Ann Arbor, MI 48109, USA; E-Mail: krullc@umich.edu

**Keywords:** chick, motor neurons, *in ovo* electroporation

## Abstract

Bidirectional signaling between Eph receptor tyrosine kinases and their cell-surface protein signals, the ephrins, comprises one mechanism for guiding motor axons to their proper targets. During projection of motor axons from the lateral motor column (LMC) motor neurons of the spinal cord to the hindlimb muscles in chick embryos, ephrin-A5 has been shown to be expressed in the LMC motor axons until they reach the base of the limb bud and initiate sorting into their presumptive dorsal and ventral nerve trunks, at which point expression is extinguished. We tested the hypothesis that this dynamic pattern of ephrin-A5 expression in LMC motor axons is important for the growth and guidance of the axons to, and into, the hindlimb by knocking down endogenous ephrin-A5 expression in the motor neurons and their axons. No perturbation of LMC motor axon projections was observed in response to this treatment, suggesting that ephrin-A5 is not needed for LMC motor axon growth or guidance.

## 1. Introduction

Proper muscle function requires that muscles be innervated by appropriate motor neurons and their axons from the central nervous system. One mechanism for guiding motor axons to their correct destination involves bidirectional signaling between the Eph family of receptor tyrosine kinases and their cell-surface protein signals, the ephrins (reviewed in [1]). Contact between a cell expressing Ephs and a cell expressing ephrins has been shown to cause repulsion between the two cells. Motor axons expressing Ephs, therefore, are thought to be guided to the appropriate destination by having all inappropriate tissues express ephrins and ensuring the appropriate tissues do not express ephrins.

**Figure 1:**
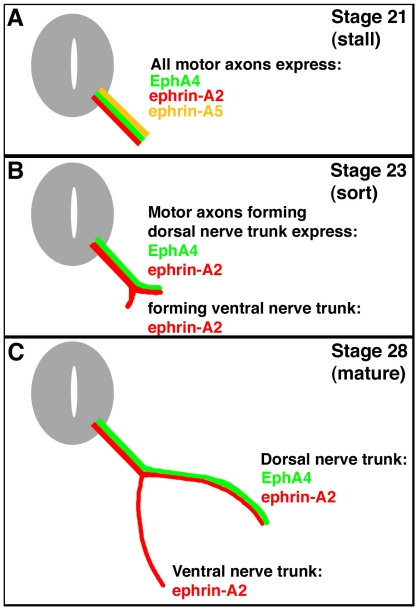
Expression of EphA4, ephrin-A5, and ephrin-A2 during motor axon projections to the chick hindlimb.

An example of this system is seen in the motor axons projecting from the lateral motor column of the spinal cord. The lateral motor column (LMC) forms at the level of the limbs and the positions of motor neurons in the LMC predicts which limb muscles they will innervate. Motor neurons and their axons in the lateral portion of the LMC project to dorsal limb muscles, whereas motor neurons in the medial portion project to ventral limb muscles [2]. It has been shown that one member of the Eph family, EphA4, is expressed only in lateral LMC motor neurons, and ephrin-A5, a member of the ephrin family of signal proteins, has its expression in ventral hindlimb mesoderm (into which muscle precursor cells migrate and develop into the ventral hindlimb muscles) [3]. Ephrin-A2 is expressed in both lateral and medial LMC motor neurons and their axons (Figure 1). There is no expression of EphAs in medial LMC neurons, nor is there expression of ephrin-As in dorsal mesoderm at this time (stage 28) [4]. Thus, lateral motor neurons of the LMC are thought to extend their motor axons only into dorsal mesoderm; these motor axons are repelled by the ephrin-A5-expressing ventral mesoderm. Medial motor neurons in the LMC are thought to extend their motor axons into the ephrin-A5 expressing ventral mesoderm, because the motor axons are thought to express no EphAs and aren’t repelled (Figure 1).

Complicating this scheme is the observation that, during their growth and initial migration out of the spinal cord, all axons of the LMC express ephrin-A5 (Figure 1) [5]. When the axons reach the base of the hindlimb and initiate sorting into their presumptive dorsal and ventral nerve trunks, this expression ceases. A summary of the expression patterns of EphA4, ephrin-A2, and ephrin-A5 on hindlimb motor axons is shown in Figure 1. Such a dynamic pattern of expression suggests that ephrin-A5 expression in the motor neurons and their axons may play a role in guiding the growth or migration of axons from the LMC prior to branching at the base of the limb bud, and/or in the limb bud after branching. Ephrin-A5 could affect growth or migration either via its receptor function, signaling back to its expressing cell, or through its ligand function, by binding to EphA4 on the surface of axons in the same tract, or both. We have tested this hypothesis by using an shRNA approach to knock down normal ephrin-A5 expression in LMC axons exiting the spinal cord. Our results show that this loss-of-function approach resulted in no motor axonal growth or migration defects.

## 2. Results and Discussion

**Figure 2:**
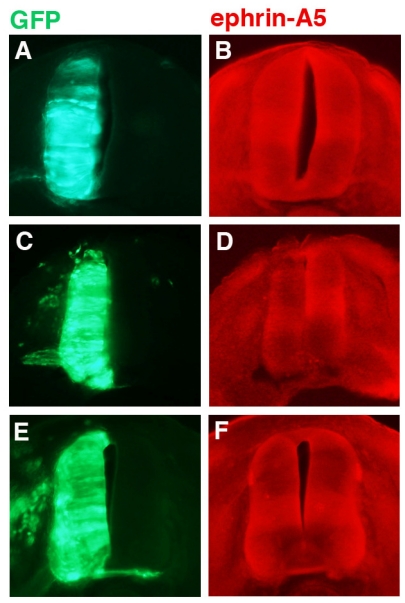
Knockdown of ephrin-A5 expression by 236 shRNA. Chick embryos were co-transfected at stage 15/16 with pCAX alone (**A**,**B**), pCAX and 236 shRNA, against ephrin-A5 (**C**,**D**), or pCAX and 236 M shRNA, a mutated form of 236 (**E**,**F**). Vibratome sections of embryos fixed at stage 21 were stained with antibody against ephrin-A5 and examined for GFP expression (**A**,**C**,**E**) or antibody staining (**B**,**D**,**F**).

Various constructs encoding ephrin-A5 shRNAs were transfected into chick neural tubes and tested for their ability to knock down ephrin-A5 expression 24 h post-transfection at stage 21; the results for one of these shRNAs (designated “236”) is shown in Figure 2. The GFP protein (green; driven by the chick beta-actin promotor and CMV enhancer) is from motor neurons transfected with either pCAX (controls), co-transfected with pCAX and shRNA against ephrin-A5 (236 shRNA), or co-transfected with pCAX and a mutated version of 236 shRNA (236 M shRNA). Stage 21 was chosen because it is a point prior to the natural down-regulation of ephrin-A5 by stage 23. For any ephrin-A5 knockdown to have an effect on LMC motor axons, it would have to be achieved while ephrin-A5 is still expressed in the LMC motor neurons, prior to or at stage 23. GFP expression indicates which cells/tissues have been transfected (Figure 2A,C,E). Expression of ephrin-A5 (Figure 2B,D,F) was observed throughout most of the neural tube, although there were areas of higher signal intensity, particularly in a horizontal stripe across the center of the neural tube, and in the motor columns. Looking at these areas, neural tubes transfected with plasmid encoding pCAX and 236 shRNA (against ephin-A5) showed decreased ephrin-A5 signal (Figure 2D) compared to control embryos (Figure 2B). In contrast, neural tubes transfected with plasmid encoding 236 M shRNA (a mutated form of 236 shRNA) exhibited no such decrease in ephrin-A5 (Figure 2F; see Experimental Section).

To assess the effectiveness of 236 shRNA, signal intensity on the transfected side of the neural tube was quantified using Image Gauge software (Fuji) and compared with signal intensity on the non-transfected side of the neural tube. Readings were taken at three spots on each side of the neural tube (two within the horizontal stripe and one within the motor column), the readings for the three spots (less background) were averaged, and the percent difference between the transfected side and the non-transfected side was calculated. The results are shown in Table 1. Embryos expressing 236 shRNA exhibited an ephrin-A5 signal intensity on the transfected side of the neural tube that was an average of more than 40% lower than on the non-transfected side of the neural tube. In contrast, control embryos (those expressing pCAX alone or pCAX with a mutated shRNA, 236 M) exhibited little difference between the two sides of the neural tube. These data suggest that 236 shRNA was effective at knocking down ephrin-A5 expression by approximately 40%, whereas 236 M shRNA was apparently ineffective. Three other shRNA constructs (targeting ephrin-A5 mRNA beginning at positions 248, 373, and 571, respectively) were also tested, but none was shown to be as effective as 236 shRNA at knocking down ephrin-A5 (data not shown). As a result, subsequent experiments were done using the 236 shRNA plasmid.

**Figure 3:**
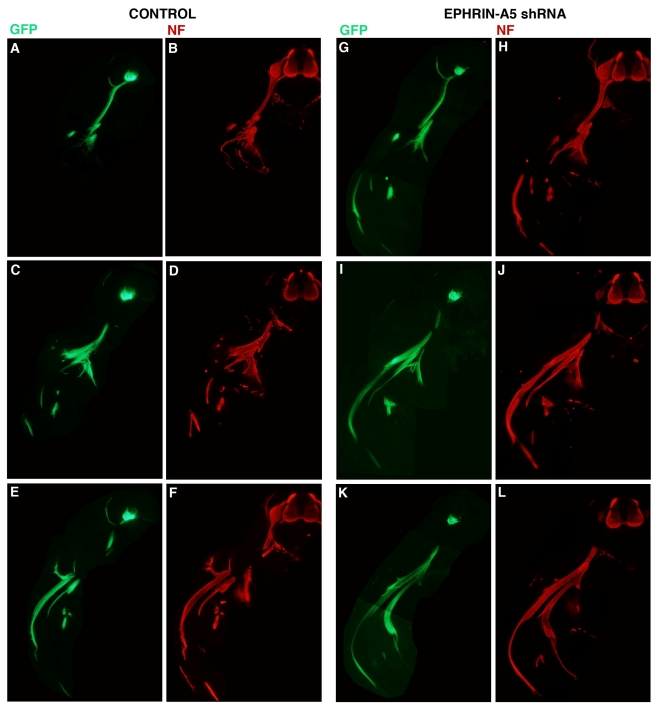
Effect of ephrin-A5 shRNA on LMC axon growth and migration. Embryos were co-transfected at stage 15/16 with HB9 and 236 M (control; **A**–**F**) or HB9 and 236 (ephrin-A5 shRNA; **G**–**L**) and fixed at stage 26. Vibratome sections were stained with anti-neurofilament antibody (NF) and examined for GFP fluorescence (**A**,**C**,**E;G**,**I**,**K**) and antibody staining (**B**,**D**,**F;H**,**J**,**L**). Note: motor axons have extended into the limb at stage 26 in **A**,**B** but are omitted here.

To assess the effect of the 236 shRNA on growth or early migration of axons from the LMC, controls and embryos transfected with plasmid encoding 236 shRNA were allowed to develop to stage 26, at which time embryos were fixed, sectioned and stained with antibodies against neurofilaments (Invitrogen; see Experimental Section). The results are presented in Figure 3. For these experiments, embryos were co-transfected with a plasmid encoding GFP under the control of the HB9 promoter, which is active only in motor neurons and their axons [6]. Three consecutive sections from representative control (Figure 3A–F; *n* = 6) and ephrin-A5 shRNA-transfected embryos (Figure 3G–L; *n* = 8) are presented, with both GFP (Figure 3A,C,E,G,I,K) and neurofilament staining (Figure 3B,D,F,H,J,L) shown for each section. GFP expression was strong in the LMC on the transfected side of the neural tubes of both control and experimental embryos, and was visible in the motor axons emanating from the LMC, confirming expression of the transfected genes in LMC neuronal cell bodies and axons. Comparison of axonal growth or migration between the two groups revealed no obvious difference between the two groups. LMC axons branched normally at the base of the limb bud in both groups (in particular, see Figure 3C/D,I/J), and extended correctly into the limb mesoderm (Figure 3E/F; Figure 3I/J,K/L). This suggests that migration of LMC axons is unperturbed by an approximate 40% reduction in ephrin-A5 expression in the neurons.

**Figure 4:**
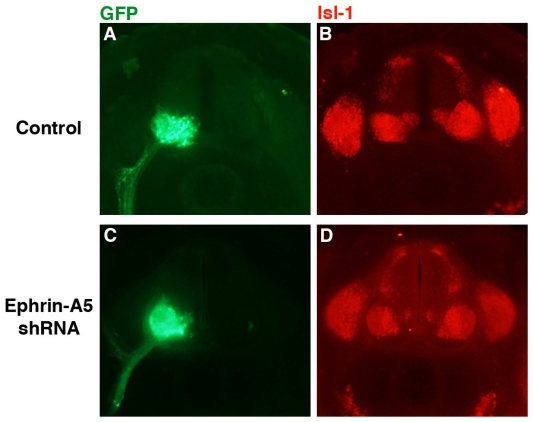
Effect of ephrin-A5 shRNA on islet-1 (isl-1) expression. Embryos were co-transfected at stage 15/16 with HB9 and 236 M (Control; **A**,**B**) or with HB9 and 236 shRNA (against ephrin-A5; **C**,**D**) and fixed at stage 26. Vibratome sections were stained with antibody against isl-1 and examined for GFP fluorescence (**A**,**C**) and antibody staining (**B**, **D**).

Despite the apparently normal projection of LMC axons, it was possible that cell bodies within the LMC sorted incorrectly or were disrupted in some manner. To examine this possibility, embryo sections were stained with antibody against islet-1 (isl-1; DSHB), which is initially expressed in all cells of the LMC, but subsequently becomes restricted to the medial LMC [7]. Sections from representative control (*n* = 6) and ephrin-A5 shRNA-expressing embryos are shown in Figure 4. GFP expression was strong in the LMC on the transfected side of the neural tube in both groups (Figure 4A,C), indicating expression of the transfected genes in the LMC and their axons. In both the control and experimental groups, isl-1 staining was observed throughout the LMC (Figure 4B,D), although expression was stronger in the medial LMC in both groups (particularly cogent in Figure 4D). These results suggest that isl-1-expressing neurons were sorting correctly within the LMC in the ephrin-A5 knockdown embryos.

## 3. Experimental Section

### 3.1. Ephrin-A5 shRNA Constructs

The chicken ephrin-A5 cDNA sequence was entered into Ambion’s siRNA Target Finder algorithm to identify potential shRNA sequences. Candidate 21-mer sequences were entered into a BLAST search to ensure no non-specific interactions with genes other than ephrin-A5. Four sequences were chosen, and shRNA sequences were devised using Ambion’s template design tool. Oligonucleotides comprising both strands of these sequences were synthesized by Invitrogen, annealed, and ligated into the Bam HI site of the pSilencer 1.0 vector (Ambion), in which shRNA transcription is driven by a U6 promoter. All plasmid constructs were sequenced by the University of Michigan sequencing core to confirm fidelity of the shRNA-encoding sequences in the vectors. The various shRNA constructs were tested by transfection into chick neural tubes at stage 15/16 using in ovo electroporation as described below, allowing the embryos to develop to stage 21, and assessing the expression of ephrin-A5. Ephrin-A5 expression was assayed by staining embryo sections with anti-ephrin-A5 antibody as described below, followed by comparison of expression on the transfected side to that on the non-transfected side (for details, see “Immunohistochemistry”, below). The shRNA that achieved the best knockdown (approximately 40% reduction in ephrin-A5 expression; see Table 1) was used for all subsequent experiments. This construct, dubbed “236 shRNA” targeted a sequence beginning at position 236 of chick ephrin-A5 mRNA (5′-AAGAUAAGACCGAACGCUAUG-3′). A mutated version of this sequence was devised by swapping positions of 4 bases within the sequence, producing a construct called “236 M shRNA”.

### 3.2. *In Ovo* Electroporation

Fertilized White Leghorn chicken eggs (MSU Poultry Farm) were incubated at 37 °C until stage 15–16 [8], windowed, and subjected to dorsoventral in ovo electroporation (targeting a ventral quadrant of the neural tube) according to procedures described previously [3,9,10]. Pulled glass micropipettes were used to introduce plasmid DNA [typically a mixture of an shRNA-encoding construct at 3–5 μg/μL and either pCAX [9] at 0.1 μg/μL or HB9-GFP [6] at 2–4 μg/μL into the neural tube, and four 15 V pulses of 50 ms duration each were delivered through home-made platinum electrodes. After electroporation, embryos were bathed in a small amount of sterile Ringer’s solution, resealed with parafilm, and incubated until reaching the desired stage.

### 3.3. Immunohistochemistry

Embryos were harvested at the desired stage and fixed overnight at 4 °C in 4% paraformaldehyde. After washing in 1X PBS, embryos were eviscerated, and the dorsal body wall and hindlimbs isolated and embedded in 6% low melting point (LMP) agarose (Sigma). 100 μm vibratome sections were prepared and stained with antibody against ephrin-A5 [4], mouse neurofilament (NF-M; Invitrogen), or islet-1 (DSHB), as described previously [3]. Appropriate Alexa Fluor 568 secondary antibodies (Invitrogen) were used to detect primary antibody binding. Antibody signal intensities were quantified using Image Gauge software (Fuji). Measurements were taken at three points each on the transfected side and the non-transfected side of each embryo, background subtracted, and comparisons were made between the two sides of the embryo.

## 4. Conclusions

Our results indicate that perturbation of normal ephrin-A5 expression in LMC motor neurons and their axons with specific shRNAs against ephrin-A5 had no effect on the growth or migration of the motor axons. Knockdown of ephrin-A5 expression using an shRNA approach did not alter the growth or migration of motor axons into the hindlimb, nor did it appear to affect sorting of the cell bodies in the LMC. Therefore, it may be that the dynamic expression pattern of ephrin-A5 is of little importance to the growth or guidance of migrating LMC axons; however, there are two possible alternative explanations.

First, it may be that ephrin-A5 expression in axons exiting the spinal cord does play a role in growth or guiding migration, but that 40% knockdown of its expression is insufficient to disrupt its function. Testing additional shRNA sequences may yield one that knocks down expression by a greater amount, although it may be that the timing of developmental events makes ephrin-A5 in LMC axons refractory to an shRNA approach. Electroporation was performed at stage 15/16 to apply the DNA to LMC precursors. Endogenous expression of ephrin-A5 ceases by about stage 23 [5], which occurs approximately 30 h beyond stage 15/16. This leaves a very narrow window in which to knock down ephrin-A5, and it is conceivable that a more robust knockdown of ephrin-A5 is not achievable within this timeframe.

Second, redundancy may have prevented our discerning a developmental role for ephrin-A5 expression in LMC axons. Ephrin-A2 is also expressed in LMC axons migrating out of the spinal cord, and this expression persists after ephrin-A5 expression is no longer detectable [4]. It is possible that persistent ephrin-A2 may be able to functionally replace the lost ephrin-A5 signaling in 236 shRNA-transfected embryos. If that is the case, it may require a double knockdown of ephrin-A5 and ephrin-A2 in order to observe disruption of growth or the normal migration pattern of LMC axons.

Taken all together, it is surprising that altering ephin-A5 function has no effect on motor axon growth or guidance to the hindlimb, using a loss-of-function approach. Perhaps ephrin-A5 contributes in some unknown manner to spontaneous activity [6]. Further electrophysiological experiments will be required to uncover the role of ephrin-A5 in motor axon growth and guidance to the hindlimb.

## Figures and Tables

**Table 1:** Percentage decrease in ephrin-A5 expression in the transfected side of the neural tube compared to the non-transfected side after in ovo electroporation with control plasmids or plasmid encoding ephrin-A5 shRNA.

Sample	pCAX only	Ephrin-A5 shRNA (236) + pCAX	Control shRNA (236 M) + pCAX
Number of embryos observed	8	9	7
Ephrin-A5 expression difference	0.45 ± 19.1%	40.42 ± 10.75%	1.62 ± 9.33%
